# Evolutionary Reduction of the First Thoracic Limb in Butterflies

**DOI:** 10.1673/031.011.6601

**Published:** 2011-05-23

**Authors:** Joanna M. Wolfe, Jeffrey C. Oliver, Antónia Monteiro

**Affiliations:** ^1^ Department of Geology & Geophysics, Yale University, New Haven, CT 06520-8109, USA; ^2^ Department of Ecology & Evolutionary Biology, Yale University, New Haven, CT 06520-8106, USA

**Keywords:** ancestral reconstruction, development, Nymphalidae, Riodinidae

## Abstract

Members of the diverse butterfly families Nymphalidae (brush-footed butterflies) and Riodinidae (metalmarks) have reduced first thoracic limbs and only use two pairs of legs for walking. In order to address questions about the detailed morphology and evolutionary origins of these reduced limbs, the three thoracic limbs of 13 species of butterflies representing all six butterfly families were examined and measured, and ancestral limb sizes were reconstructed for males and females separately. Differences in limb size across butterflies involve changes in limb segment size rather than number of limb segments. Reduction of the first limb in both nymphalids and riodinids appears particularly extensive in the femur, but the evolution of these reduced limbs is suggested to be a convergent evolutionary event. Possible developmental differences as well as ecological factors driving the evolution of reduced limbs are discussed.

## Introduction

Arthropods display remarkable diversity in their segmented body plans, including variation in the number, shape, and size of their limbs. Recent studies have examined both the evolution of changes in arthropod limb number (Adamowicz and Purvis 2006), as well as more subtle changes in limb segment sizes (Mahfooz et al. 2004, 2007), focusing on crustacean and insect limbs, respectively. However no study has focused on the quantitative evolution of limbs within butterfly species.

**Figure 1.**
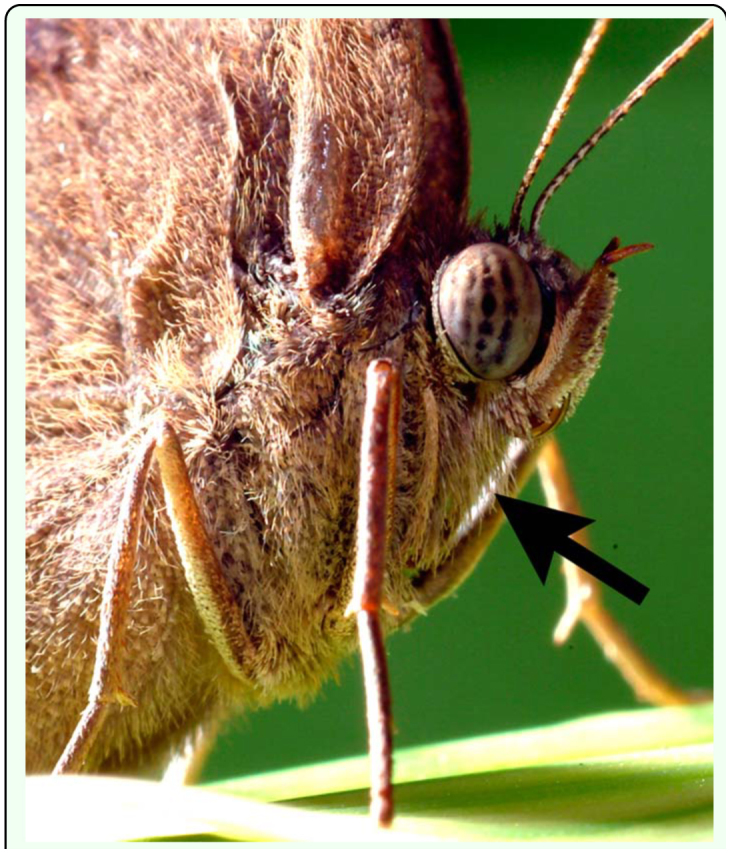
Reduced first thoracic limbs of *Bicyclus anynana* (Nymphalidae). Indicated by the black arrow. Photo courtesy of W. Piel. High quality figures are available online.

Two butterfly families, the Nymphalidae and Riodinidae, have reduced T1 limbs that are not used for walking. Reduced T1 limbs are likely a derived trait, as basal families such as the Papilionidae and Pieridae, as well as most other insects, have three pairs of walking legs (Fox 1966). The family Nymphalidae, one of the most diverse lineages of butterflies, comprises about 6000 species, including common species such as the Monarch, morphos, painted ladies, and emperors (Ackery et al. 1999). The reduced T1 limb is usually covered in dense hairs, giving the family the common name “brush-footed butterflies” (Figure 1) (Fox 1966). The Riodinidae, comprising about 1000 species, are a sister lineage to the Lycaenidae, and both these lineages are sister to Nymphalidae. The T1 limbs of riodinids, but not those of lycaenids, also appear reduced (Robbins 1988). Since no detailed measurements of the distal limb segments (i.e. the femur, tibia and tarsus) are available for any of the families, it is unclear whether limb reductions seen in Nymphalidae and Riodinidae were due to a single event or were the result of convergent evolution.

Here, a pilot analysis of limb size evolution in butterflies across all families is presented. How (in which limb segments) and when (phylogenetically) reduction has occurred is examined. Two processes of morphological change could result in an overall reduction in the T1 limb. Limb segments might be reduced by an equal proportion, making the total limb length shorter. Alternatively, limb length differences may be caused by a disproportionate reduction in particular limb segments. The ancestral states of all limbs in butterflies were estimated, to determine when limb reduction may have occurred in the evolution of butterflies.

To determine how and when limb reduction took place in butterflies, all leg segments were measured in both sexes across a variety of species spanning all five families of Papilionoidea (Papilionidae, Pieridae, Nymphalidae, Lycaenidae, and Riodinidae) and the out-group Hesperiidae. The ancestral size of each limb segment was then estimated for males and females separately based on the phylogeny, as it is possible that limbs have evolved differently in each sex. Branches where significant shifts in limb size took place were identified by estimating the ancestral states for adjacent nodes. Finally, possible developmental mechanisms underlying limb reduction as well as the ecological function of reduced first thoracic limbs are discussed.

## Materials and Methods

### Species studied

Limb morphology was studied in 13 species of butterflies, representing all six families: *Epargyreus clarus* (Hesperiidae: Pyrginae), *Lycaena phlaeas* (Lycaenidae: Lycaeninae), *Satyrium titus* (Lycaenidae: Theclinae), *Asterocampa clyton* (Nymphalidae: Apaturinae), *Bicyclus anynana* (Nymphalidae: Satyrinae), *Junonia coenia* (Nymphalidae: Nymphalinae), *Libytheana carinenta* (Nymphalidae: Libytheinae), *Parnassius phoebus* (Papilionidae: Parnassiinae), *Neophasia terlooii* (Pieridae: Pierinae), *Pieris rapae* (Pieridae: Pierinae), *Zerene eurydice* (Pieridae: Coliadinae), *Apodemia mormo* (Riodinidae: Riodininae), and *Calephelis borealis* (Riodinidae: Riodininae). Although not all subfamilies are represented, most of the highly species-rich ones have been included (with the exception of the nymphalid subfamily Heliconiinae) (Brower 2008). One male and one female of each species were studied. Specimens housed in the Yale Peabody Museum collections were used, with the exception of *B. anynana* and *P. rapae,* which were reared in the laboratory.

### Dissections and limb measurements

For each individual, the specimen was pinned ventral side up, and one limb from each thoracic segment (T1, T2, and T3) was dissected under a Carl Zeiss Discovery.V8 stereomicroscope at 40x magnification. Dissected limbs were photographed using an AxioCam camera controlled by AxioVision AC v.4.5.0.0 software. Limb segment lengths were measured using ImageJ (Abramoff et al. 2004), by defining the x,y coordinate points at the end of each segment and later calculating the length between points. The femur, tibia, and tarsus were measured from proximal to distal. Pairwise length comparisons across thoracic limbs were made by plotting the length of each limb segment (and the entire limb) of T1 vs. T2, T1 vs. T3, and T2 vs. T3.

### Phylogenetic analysis

Relationships among taxa included in this study were based on the results of Wahlberg et al. (2005; as reported in Brower 2008). This topology was used to estimate limb character evolution in Mesquite (Maddison and Maddison 2009). The ancestral lengths of four continuously coded traits were reconstructed: femur, tibia, combined tarsus, and total limb length. Ancestral character states were reconstructed for each node using squared-change parsimony, which estimates the ancestral states by minimizing the squares of the difference between ancestor and descendant (Maddison 1991). Reliable estimates of branch lengths were not available for the tree used in reconstructions, so changes were not weighted by branch lengths.

In order to identify when limb reductions may have occurred, a reduction threshold based on limb size relationships of contemporary taxa was used. In all nymphalid species and one riodinid species included in this study, the length of T1 limbs measured less than 80% of the average length of the T2 and T3 limbs (see Results). This threshold, 80% of the average length of T2 and T3, was used as a conservative, but arbitrary, threshold to identify significant limb reduction events on the phylogeny. Such events were inferred to occur when a descendant, with whole limb or limb segment meeting the 80% reduction threshold, arose from an ancestor that did not meet the reduction threshold (i.e. the ancestral state of the whole limb or limb segment was ≥ 80% of the average length of the corresponding segment in T2 and T3).

**Figure 2.**
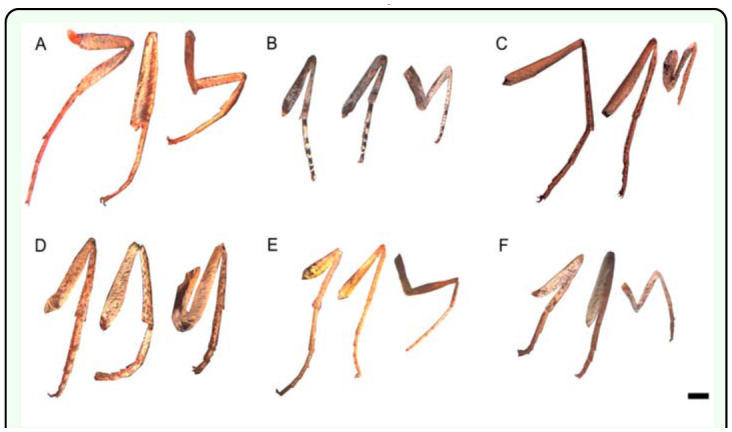
Photographs of limbs (from left: T3, T2, T1) from representative male individuals of (a) *Epargyreus clams* (Hesperiidae), (b) *Satyrium titus* (Lycaenidae), (*c*) *Junonia coenia* (Nymphalidae), (d) *Parnassius phoebus* (Papilionidae), (e) *Zerene eurydice* (Pieridae), and (f) *Apodemia mormo* (Riodinidae). Scale bars = 1 mm. Note marked reduction of T1 limbs in (c) and (f). High quality figures are available online.

## Results

**Figure 3.**
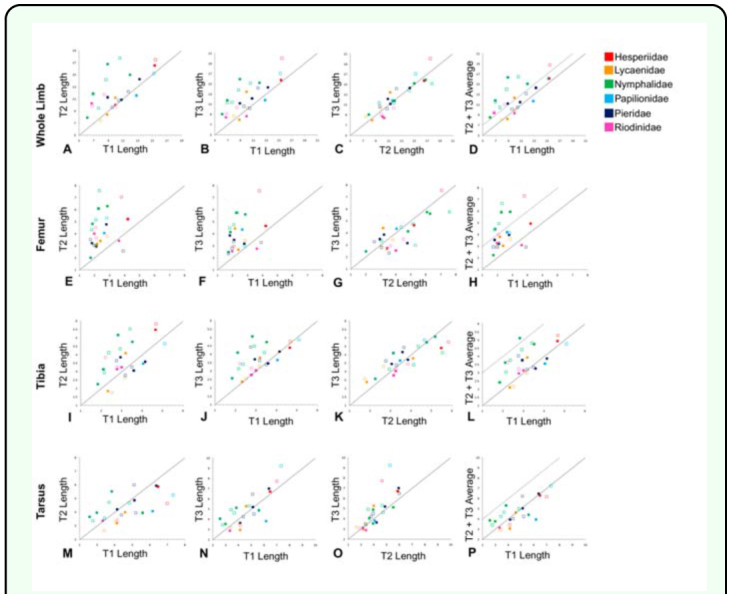
Plots of limb segment lengths. All scales in millimeters. The solid line in all plots marks isometry, so points that fall above the line are smaller on the x-axis than on the y-axis. (a)-(d): whole limb lengths, (e)-(h): femur, (i)-(l): tibia, (m)-(p): tarsus (all S segment lengths combined). Filled squares represent males, open squares represent females. Colors represent different butterfly families. Dashed line indicates the 80% reduction threshold. High quality figures are available online.

Representative photographs of male limbs are shown in Figure 2. Note the relative size difference of T1 in the nymphalid *J. coenia* (Figure 2c), and the riodinid *A. mormo* (Figure 2f), compared with the other families. True thoracic limbs were present in all species, though all nymphalids and riodinids hold their T1 limbs against the body. All studied members of the family Nymphalidae (in green, Figure 3a–c), *C. borealis* (Riodinidae, in pink), and the female *A. mormo* (Riodinidae) had T1 limbs that were less than 80% of the average length of T2 and T3. The relationship between T2 and T3 across species is close to isometry, with major exceptions in the *A. clyton* female, *C. borealis* male (both with T2 larger than T3), and *P. phoebus* female (T3 larger than T2).

**Figure 4.**
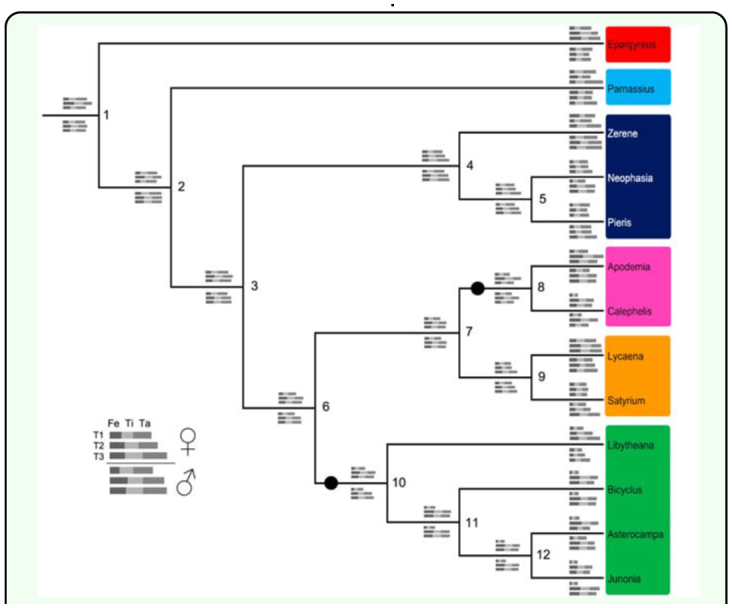
Cladogram of included species based on the topology in Wahlberg et al. (2005). Grey bars at nodes show inferred ancestral states for lengths of femur, tibia, and total (all 5) tarsus for all three limbs of both sexes. Node numbers refer to ancestral state estimates shown in Table 1. Estimates of females limbs are above the branches, male limbs are below. For each species, limb lengths are scaled relative to the length of the male T3 limb. The T1 femur is inferred to be reduced at both the base of the Nymphalidae and the base of the Riodinidae (indicated by black circles on branches). Families are designated by colored boxes around genus names (see legend in Figure 3). High quality figures are available online.

In these reconstructions of limb evolution, the T1 limb appears to have evolved a reduced morphology independently in the ancestors of the Nymphalidae and Riodinidae (Figure 4 and Table 1), as the common ancestor of both families (also the common ancestor of Lycaenidae) did not share the reduced T1 limb morphology based on the threshold measure. These convergent reductions occurred at the base of the Nymphalidae, and at the base of the Riodinidae (black circles in Figure 4). In both cases, the reduction is particularly evident in the T1 femur (Figure 3d, f). T1 reduction is also seen to some extent in the tarsus, but not in the tibia, suggesting the reduction occurred primarily in a subset of limb segments (femur and tarsus) and not across the whole limb.

## Discussion

In the sample of thirteen species spanning all butterfly families, both Nymphalidae and Riodinidae were found to have reduced T1 limbs. Interestingly, the estimates of limb evolution suggest that the same limb segments (femur and tarsus) were reduced convergently in members of these two families. As ancestral reconstructions are estimates of the most likely evolutionary events, future studies are necessary to determine the plausibility of alternative models of limb reduction in butterflies. For example, it is possible that the T1 limb reduction occurred in the lineage that gave rise to the Nymphalidae, Riodinidae, and Lycaenidae, followed by a secondary lengthening of the T1 in the ancestor to Lycaenidae. The taxon sampling in this study is relatively sparse, and should be expanded by at least double or triple in order to further support these preliminary conclusions. Basal lycaenid genera of particular interest include *Curetis* (Lycaenidae: Curetinae), *Miletus* (Lycaenidae: Miletinae), and *Poritia* (Lycaenidae: Poritiinae). These taxa could provide further information about the ancestral state of the common ancestor of Nymphalidae, Riodinidae, and Lycaenidae. If lycaenids included in future analyses display reduced T1 limbs, this would suggest that limb reductions happened only once at the base of all three families.

**Table 1.**
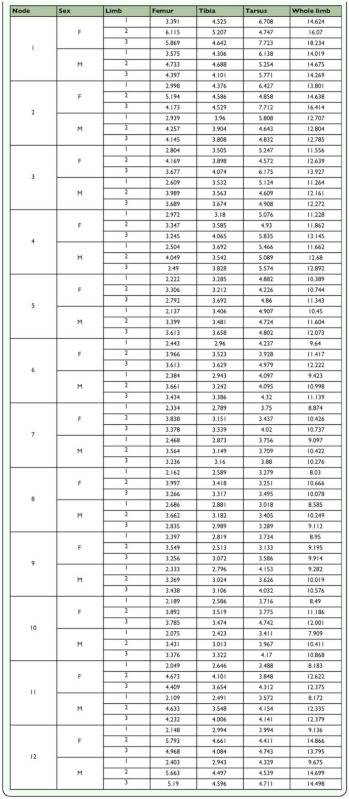
Ancestral whole limb and limb segment lengths estimated using squared-change parsimony in Mesquite (Maddison and Maddison 2009). Numbers refer to nodes of the cladogram in Figure 4.

Discernment among hypotheses of limb reduction will also require a better understanding of the developmental mechanisms underlying current patterns of variation in limb sizes of Lepidoptera. The developmental basis of divergent limb morphologies along the anterioposterior body axis has been examined, to some extent, in crustaceans and some insect lineages, but not in the ventral appendages of butterflies. Based on developmental experiments in extant arthropod taxa, the morphology of the serially homologous limbs is proposed to be affected by the following mechanisms: anterioposterior shifts in Hox gene expression domains changing the regulation of particular sets of limbs (Averof and Patel 1997; Abzhanov and Kaufman 1999; Pavlopoulos et al. 2009), changes in limb developmental genes to make them sensitive to conserved patterns of Hox gene regulation (Tomoyasu et al. 2005; Warren et al. 1994), and the appearance of novel Hox gene expression domains that change the size of particular limb segments (Khila et al. 2009; Mahfooz et al. 2004, 2007; Stern 2003).

From the different mechanisms outlined above, the novel expression of Hox genes in spatially restricted domains within a limb is the only mechanism that has been associated with changes in ventral limb size in insects. Mahfooz et al. (2004) discovered that expression of the Hox gene *Ultrabithorax* (*Ubx*) was quantitatively correlated to the allometric enlargement of T3 limbs in diverse hemimetabolous insect taxa, such as grasshoppers and cockroaches. Functional experiments showed that *Ubx* promotes leg growth in the regions where it is expressed (Mahfooz et al. 2007). In water striders (Hemiptera), T2 limbs are the largest of the thoracic limbs and Ubx expression is found there, as well as in T3 limbs (Khila et al. 2009). Functional experiments also determined that *Ubx* increases the length of T2 limbs, but reduces the size of T3 limbs (Khila et al. 2009), suggesting additional complexity in the underlying regulatory network where *Ubx* can act as either an enhancer or repressor of growth. Although *Ubx* expression domains are not yet known from T1 limbs in butterflies nor T1 limbs in any other insect, a change in the regulation of *Ubx* (or possibly other Hox genes) could be involved in the allometric reduction of nymphalid and riodinid T1 legs.

The ecological function of reduced T1 limbs in butterflies is primarily sensory. In all the species studied here, is possible that a subset of T1 hairs, the sensillae, are involved in particular chemo- or mechano-sensory functions (Fox 1966). The T1 tarsi of nymphalids, for instance, show “negative” (unpleasant) reactions to sugar solutions, while the T2 and T3 tarsi show positive reactions (Fox 1966). In contrast, both T1 and T2 of pierids had positive reactions to sugar solutions (Fox 1966). Female nymphalids use sensillae distributed on tarsi of the T1 leg to taste the host plant prior to oviposition. This has been observed in behavioral studies (Myers 1969; reviewed in Renwick and Chew 1994). Further experiments tested the necessity of tarsal sensillae for oviposition (Calvert and Hanson 1983) and physiological reactions of sensillae to compounds from their own versus other host plants (Baur et al. 1998), confirming the tarsi are used for host plant recognition. The chemosensory differentiation of T1 relative to the other limbs in nymphalids suggests that they evolved a new chemosensory function whilst losing their walking function. Currently, no sensory data are available for riodinids, and it is unclear whether the limb reductions here are also associated with similar T1–T2 sensory differentiation as seen in nymphalids. To draw further conclusions it will be necessary to carry out detailed morphological studies (such as SEM) of the tarsal structures of riodinids and nymphalids and study their neurobiological function. In addition, detailed behavioral observations are necessary to understand the function of T1 limbs in these lineages.

## Conclusions

In the first systematic study of limb evolution in butterflies, it is shown that members of the families Nymphalidae and Riodinidae likely evolved reduced forelimbs in parallel. The reduction is particularly evident in the femur, the length of which is controlled by Hox gene expression in other insects (Mahfooz et al. 2004, 2007; Stern 2003). Future work should include more comprehensive taxon sampling, to test our assertion of convergent limb reduction in two families. Gene expression studies may also provide a mechanistic understanding for the specific reduction of the femur.

## **Abbreviations**

**MP**,: maximum parsimony;
**T1**,: first thoracic limb;
**T2**,: second thoracic limb;
**T3**,: third thoracic limb;
***Ubx***,: *Ultrabithorax*
